# Mitochondrial Dysfunction Correlates with Brain Amyloid Binding, Memory, and Executive Function in Down Syndrome: Implications for Alzheimer’s Disease in Down Syndrome

**DOI:** 10.3390/brainsci15020130

**Published:** 2025-01-28

**Authors:** Jessica A. Beresford-Webb, Catherine J. McAllister, Alison Sleigh, Madeleine J. Walpert, Anthony J. Holland, Shahid H. Zaman

**Affiliations:** 1 Cambridge Intellectual and Developmental Disabilities Research Group, Department of Psychiatry, University of Cambridge, Douglas House, Trumpington Road, Cambridge CB2 8AH, UK; 2 Wolfson Brain Imaging Centre, University of Cambridge and NIHR Cambridge Clinical Research Facility, Cambridge University Hospitals NHS Foundation Trust, Institute of Metabolic Science, Cambridge Biomedical Campus, Cambridge CB2 0QQ, UK; 3 Cambridgeshire & Peterborough Foundation NHS Trust, Douglas House, Trumpington Road, Cambridge CB2 8AH, UK

**Keywords:** Down syndrome, Alzheimer’s disease, dementia, mitochondria

## Abstract

**Background/Objectives:** Mitochondrial dysfunction is increasingly recognized as a central contributor to neurodegenerative diseases and age-related cognitive decline. Individuals with Down syndrome (DS) are at high risk of neurodegeneration due to Alzheimer’s disease (AD). This study aims to explore the relationship between mitochondrial dysfunction, brain amyloid-beta (Aβ) deposition, and cognitive decline in this population. **Methods:** We investigated mitochondrial function, brain amyloid-beta burden, and cognitive performance in a pilot study of a cohort of 10 eligible adults with DS selected from a sample of 28 individuals with DS. Phosphorus-31 magnetic resonance spectroscopy (^31^P-MRS) was used to assess mitochondrial function in skeletal muscle using a post-exercise paradigm, while positron emission tomography using ^11^C-Pittsburgh compound B (PiB-PET) measured brain Aβ deposition. Cognitive performance was evaluated using the Cambridge Cognitive Examination adapted for individuals with Down syndrome (CAMCOG-DS) and executive function batteries. **Results:** Significant correlations were observed between slowed phosphocreatine (PCr) recovery in muscle and increased Aβ deposition in key brain regions, particularly the striatum. Cognitive performance inversely correlated with mitochondrial function, with pronounced deficits in memory and executive function tasks. Notably, an individual carrying the APOE-ε4 allele exhibited the poorest mitochondrial function, highest Aβ burden, and most severe cognitive impairment, suggesting a potential interaction between genetic risk and mitochondrial health. **Conclusions:** These findings highlight the role of mitochondrial dysfunction in DS-associated AD (DSAD) and its impact on cognition in adults. The results support targeting mitochondrial pathways as a potential therapeutic strategy to mitigate AD progression in DS populations. Further research with larger cohorts and longitudinal designs is needed to clarify causative mechanisms and develop effective interventions.

## 1. Introduction

Mitochondria are conserved organelles that generate adenosine triphosphate (ATP) through oxidative phosphorylation to support neuronal homeostasis and function, play roles in biosynthesis, calcium regulation during signal transduction, and presynaptic transmitter synthesis, and act as a hub for cell survival and stress regulation [1]. Playing a central role in numerous cellular processes, mitochondrial dysfunction is particularly impactful on brain health due to the organ’s high energy demands and is increasingly recognized as a key contributor to the pathophysiology of neurodegenerative diseases, including Alzheimer’s disease (AD) [2,3,4,5,6].

To date, a large body of research has highlighted the critical role of mitochondrial dysfunction in AD pathogenesis. The “mitochondrial cascade hypothesis” has been proposed to explain AD positing that mitochondrial dysfunction initiates pathological processes, including amyloid precursor protein (APP) dysregulation, amyloid-beta (Aβ) accumulation, and oxidative damage, which drive the onset and progression of AD [5,7]. Since the mitochondrial cascade hypothesis was proposed, the link between mitochondrial dysfunction and AD pathology has been shown particularly in mice models, with a decrease in mitochondrial function leading to an increase in amyloid plaques. [8]. Moreover, Aβ and tau have been shown to exacerbate mitochondrial dysfunction, creating a feed-forward loop that accelerates neurodegeneration [5,6,9,10,11]. Studies identifying mitochondrial abnormalities in the brains of AD patients have also shown that impaired energy metabolism often precedes clinical symptoms [5,6].

The importance of mitochondrial function in AD is further evidenced by its intersection with genetic risk in AD. In transgenic models overexpressing APP, resulting in increased Aβ production, studies have confirmed that APP is targeted to neuronal mitochondria, disrupting their function [12]. Additionally, the presence of apolipoprotein E (APOE) ε4 (the variant of the apolipoprotein E (APOE) gene and the strongest genetic risk factor for AD [13]) has been associated with reduced mitochondrial complex activity and altered mitochondrial protein expression [14,15,16], suggesting that APOE genotype modulates mitochondrial function.

People with Down syndrome (DS) are at an exceptionally high risk of developing AD due to the triplication of chromosome 21 (Hsa21), which contains the APP gene, resulting in increased Aβ production [17,18,19]. Moreover, mitochondrial dysfunction, resulting from overexpression of several Hsa21 genes involved in mitochondrial function [20], has been shown to manifest systemically, and especially in people with DS, with many of the clinical features of DS overlapping with ‘mitochondrial myopathies’ including low muscle strength, obesity, developmental delay, visual deficits, hearing impairments, seizures, and poor gait control and coordination [21,22,23,24]. Despite the heightened risk of AD and evidence of mitochondrial defects, little is known about how mitochondrial dysfunction contributes to cognitive decline with age and to DSAD progression. Whilst some studies show a correlation between mitochondrial DNA mutations and amyloid pathology in DS post-mortem brains [4,25,26], there are little data available investigating mitochondrial function in vivo and its relationship to Alzheimer’s disease pathology in vivo in people with DS.

This study was designed as a pilot study with the intention of carrying out analyses that might indicate the direction of future studies and the ways in which mitochondrial function may be involved in DSAD. The approach was broadly segregated into understanding mitochondrial relationships with pathology, namely, brain amyloid deposition, and cognition in individuals with DS. Broadly, we expect that higher mitochondrial function will be associated with lower amyloid binding and higher cognitive performance. Specifically, we sought to achieve the following:

- Examine the relationship between mitochondrial dysfunction in skeletal muscle, as measured by ^31^P-MRS, and brain amyloid deposition, as assessed by PiB-PET imaging.
- Explore correlations between mitochondrial dysfunction in muscle and cognitive performance using cognitive assessments.

## 2. Materials and Methods

### 2.1. Ethics

This study was designed to run concurrently with an overarching study of dementia in people with DS, known as the Defeat Dementia in Down syndrome (DiDs) study. The data included in the DiDs study comprised structural magnetic resonance imaging (MRI), amyloid deposition measures quantified by carbon-11-labelled Pittsburgh compound B ([^11^C] PiB) positron emission tomography (PET), information regarding the clinical status of dementia, and the results of a battery of neuropsychological tests of cognitive function.

Ethical approval was granted for the study protocol by the National Research Ethics Committee (NRES Committee East of England—Cambridge Central; reference, 12/EE/0249) and for the positron emission tomography (PET) brain scans by the Administration of Radioactive Substances Advisory Committee. Written informed consent was obtained from all participants with DS who had the capacity to consent. For those without capacity to consent, the procedures specified in the England and Wales Mental Capacity Act (2005) or the Adults with Incapacity Act (Scotland Act) were adhered to.

### 2.2. Participants

Participants of the DiDS study were recruited through the Down Syndrome Association UK (DSA). Individuals who were over the age of 18 years, had participated in the DiDS study, and who had consented to being contacted about future studies were invited to participate in the current study. Invitation letters included a reply slip for participants to indicate interest and provide the name and contact details of a primary informant (the “person who knows him/her best”). Home visits were subsequently arranged to discuss the study procedures in detail and to obtain informed consent. The exclusion criteria were hypothyroidism, active cardiac disease, diabetes mellitus, pregnancy, smoking, or contraindications for MRI.

### 2.3. ^31^P Magnetic Resonance Spectroscopy (^31^P-MRS)

Participants were asked to refrain from exercise and alcohol for at least 24 h, from caffeine on the day of the scan, and from food for 2 h prior to the scan. During a home visit, DS participants were shown a short film demonstrating the in-scanner exercise to familiarise them with what would be asked of them during the scan and to convey the dimensions of the scanner. Further MRS practice sessions took place with the DS group at the NIHR Cambridge Clinical Research Facility, simulating the in-scanner exercise and accompanied by an audio file of exercise instructions which was used during the actual scans.

^31^P-MRS data were acquired on a 3 Tesla Siemens MAGNETOM Verio scanner (Erlangen, Germany). During scanning, participants lay supine with a nine-centimetre surface coil placed on the right quadricep and a weight fastened to the right ankle. Individual weights were calibrated for each participant based on their peak torque, measured with a dynamometer, to achieve a 20–30% reduction in phosphocreatine (PCr) levels post-exercise [27]. This decrease was sufficient to capture alterations in muscle signal while minimizing the risk of acidosis.

Participants entered the scanner feet first and completed a 12-min exercise sequence, raising and lowering their leg. The exercise protocol consisted of cycles of 1 min of rest, 1 min of exercise, and 4 min of rest, repeated twice [28,29,30]. This enabled two measurements of the post-exercise PCr recovery half-time, which were averaged for analysis. If any technical issues or deviations from the protocol occurred, a third session was conducted.

Spectra were analysed using jMRUI software V3.0 [31] and fitted using the AMARES algorithm [32], consistent with prior studies [28,29,30]. The PCr recovery half-time (t_1/2_), which is inversely proportional to mitochondrial oxidative capacity, was found by fitting the PCr recovery time to a two-parameter monoexponential fit.

### 2.4. Brain Imaging

Brain imaging data collection and analysis followed protocols from the DiDs pilot study [33]. [^11^C]PiB was synthesized at the Wolfson Brain Imaging Centre radiochemistry laboratory with high radiochemical purity (>95%) and specific activity (>150 GBq/μmol) under Good Manufacturing Practices. Structural MRI scans were conducted on the same 3 Tesla Siemens MAGNETOM Verio scanner (Siemens Healthineers, Erlangen, Germany) used for ^31^P-MRS imaging. Co-registration of MRI and PET data facilitated the identification of regions of interest (ROIs), and a 90 min dynamic PiB PET scan was performed using a GE Advance PET scanner (GE Medical Systems, Chicago, IL, USA). Binding potential (BP_ND_) maps were generated with the simplified reference tissue model using grey matter tissue from the cerebellum as a reference [34]. Image co-registration and manual ROI selection were conducted using SPM8 software (www.fil.ion.ucl.ac.uk/spm/software/, accessed on 1 September 2013).

### 2.5. Cognitive Assessment in DS

Cognitive function in DS participants was assessed using the Cambridge Examination for Mental Disorders of Older People with Down syndrome and Others with Intellectual Disabilities (CAMDEX-DS) [35]. This includes both a cognitive assessment of the participant (CAMCOG-DS) and an informant interview completed with a caregiver evaluating functional decline over time.

The quantitative cognitive scores reported here are derived from the CAMCOG-DS, which assesses seven cognitive domains: orientation, language, memory, attention, praxis, abstraction, and perception. Dementia status was derived from the CAMDEX-DS informant interview, independently assessed by a blinded psychiatrist.

### 2.6. Executive Function Battery

Collected as part of the DiDS study, executive function was evaluated using the Cambridge Executive Function Battery [36]. Table 1 outlines the tasks used, each linked to specific cognitive processes. See Appendix A.1.1, Appendix A.1.2, Appendix A.1.3, Appendix A.1.4, Appendix A.1.5 and Appendix A.1.6 for descriptions of the tasks used.

### 2.7. Genetics and Biochemistry

Blood samples were analysed at the National Institute for Health Research Cambridge Biomedical Research Centre Core Biochemistry Assay Laboratory. APOE allelic status was determined using the Cambridge University Hospitals Biomedical Campus standard protocols. DNA was extracted from peripheral blood by technicians who were blinded to clinical and biomarker data, and APOE genotyping was determined by polymerase chain reaction amplification.

### 2.8. Statistical Analysis

Data normality and homogeneity of variance were verified using Kolmogorov–Smirnov and Levene’s tests, respectively, in IBM SPSS software V22.0 (SPSS Inc., Chicago, IL, USA). The specific statistical tests used for each comparison are specified in the results. Descriptive statistics are presented with standard deviations, and significance was set at *p* < 0.05 with two-tailed tests unless otherwise noted.

## 3. Results

### 3.1. Participant Characteristics

A total of 28 individuals with Down syndrome (DS) participated in the MRS scanning sessions. Two individuals were excluded prior to scanning: one due to a previously undisclosed metal contraindication and another due to claustrophobia. Six additional participants were excluded during data analysis because of low signal-to-noise ratios attributed to high adipose tissue levels or insufficient PCr level drops due to task motivation. Ultimately, 20 participants with analysable data were included, and among these, amyloid binding and cognitive data were available for 10 participants (Table 2).

### 3.2. Correlation of PCr Recovery Time with Amyloid Binding in DS

Two-tailed correlation analyses was conducted for MRS data and amyloid binding levels. For the binding datasets that did not fulfil the criteria for normality assumptions, nonparametric Spearman’s rho assessments were used. Pearson’s correlations were used for frontal and hippocampal correlations with PCr recovery half-time.

**Figure 1:**
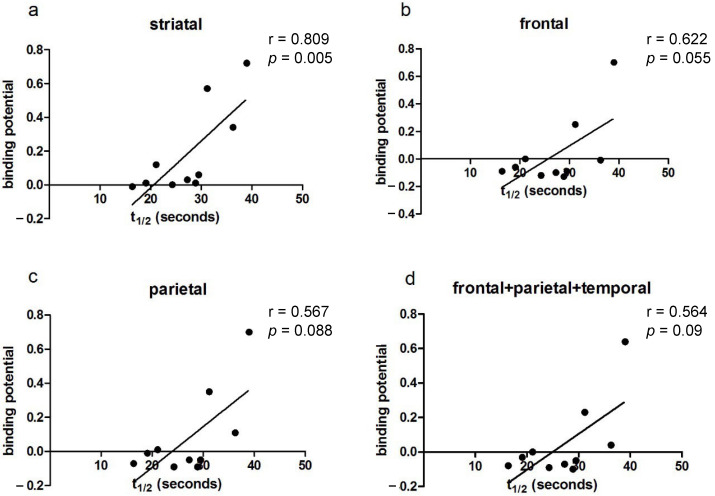
Correlations of PCr recovery half-time (t_1/2_) with amyloid binding in (**a**) striatal, (**b**) frontal, (**c**) parietal, and (**d**) frontal + parietal + temporal brain regions in DS participants, showing statistical significance for striatal binding. Each data point represents an individual participant.

A significant positive correlation was observed in the striatal region between amyloid binding and PCr recovery half-time (r = 0.809, *p* = 0.005; Figure 1a). A trend toward significance was observed in the frontal (r = 0.622, *p* = 0.055; Figure 1b) and parietal (r = 0.567, *p* = 0.088; Figure 1c) regions, as well as for combined frontal, parietal, and striatal binding (r = 0.564, *p* = 0.09; Figure 1d). No significant relationship was found for hippocampal binding (*p* = 0.391)

### 3.3. Cognitive Function and PCr Recovery Half-Time

**Figure 2:**
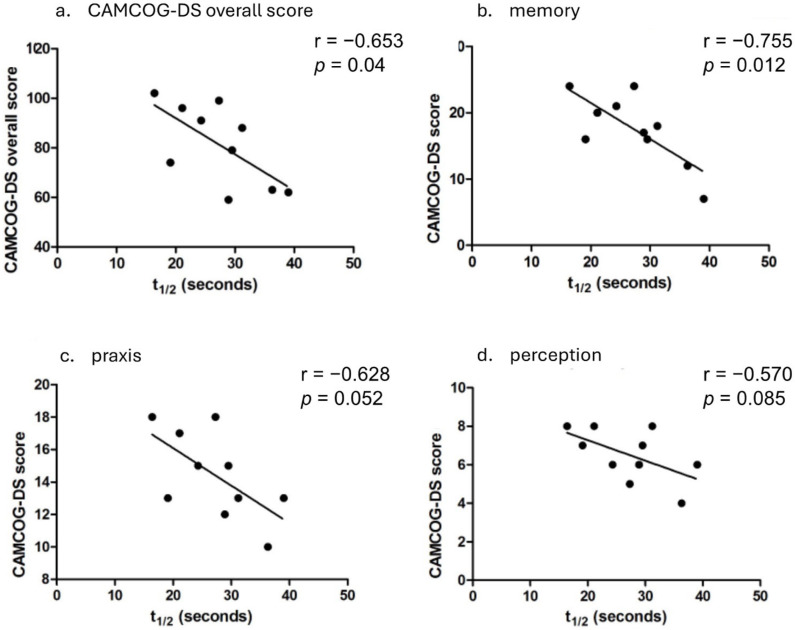
Relationship between mitochondrial function (PCr recovery half-time, t_1/2_) and CAMCOG-DS total (**a**), memory (**b**), praxis (**c**), and perception (**d**) scores, showing significant or trending associations. Each data point represents an individual participant.

CAMCOG-DS total scores ranged from 59 to 102 (out of a possible 109). Linear regression analysis indicated a strong negative association between PCr recovery half-time and CAMCOG-DS total scores (r = −0.653, *p* = 0.04, Figure 2). When age effects were controlled, PCr recovery half-time accounted for 60% of the variation in CAMCOG performance, though the correlation did not reach statistical significance (r = −0.6, *p* = 0.088).

PCr recovery half-time significantly correlated with CAMCOG-DS memory domain scores (r = −0.755, *p* = 0.012; Figure 2b) and showed a trend toward correlation in praxis (r = −0.628, *p* = 0.052; Figure 2c) and perception (r = −0.570, *p* = 0.085; Figure 2d) domains. No significant associations were found with attention (*r* = −0.495, *p* = 0.145) or abstract thinking (r = −0.348, *p* = 0.324).

**Figure 3:**
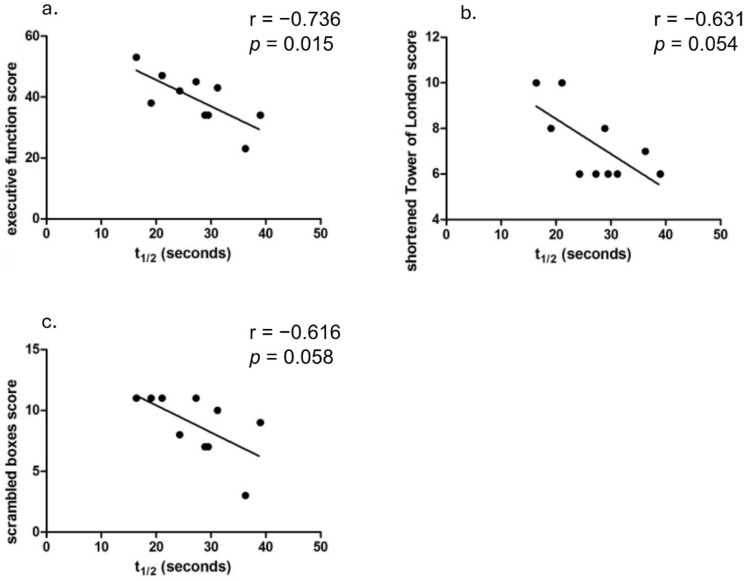
Correlations between PCr recovery half-time (t_1/2_) and executive function measures in DS participants, showing significant or trending associations with overall battery score (**a**), Tower of London (**b**), and Scrambled Boxes (**c**) tasks. Each data point represents an individual participant.

For executive function, Pearson’s correlation analysis showed a strong relationship between PCr recovery half-time and total executive function battery score (r = −0.736, *p* = 0.0153; Figure 3a). Significant correlations were also observed with the Tower of London task (r = −0.631, *p* = 0.05; Figure 3b) and there was a trending association with the Scrambled Boxes task (r = −0.616, *p* = 0.058; Figure 3c).

### 3.4. Case Studies of APOE Allelic Status

Among the 10 participants, APOE genotyping data were available for 9 individuals. Of these, one participant had an Ɛ4 allele, and another had an Ɛ2 allele (Table 3).

**Figure 4:**
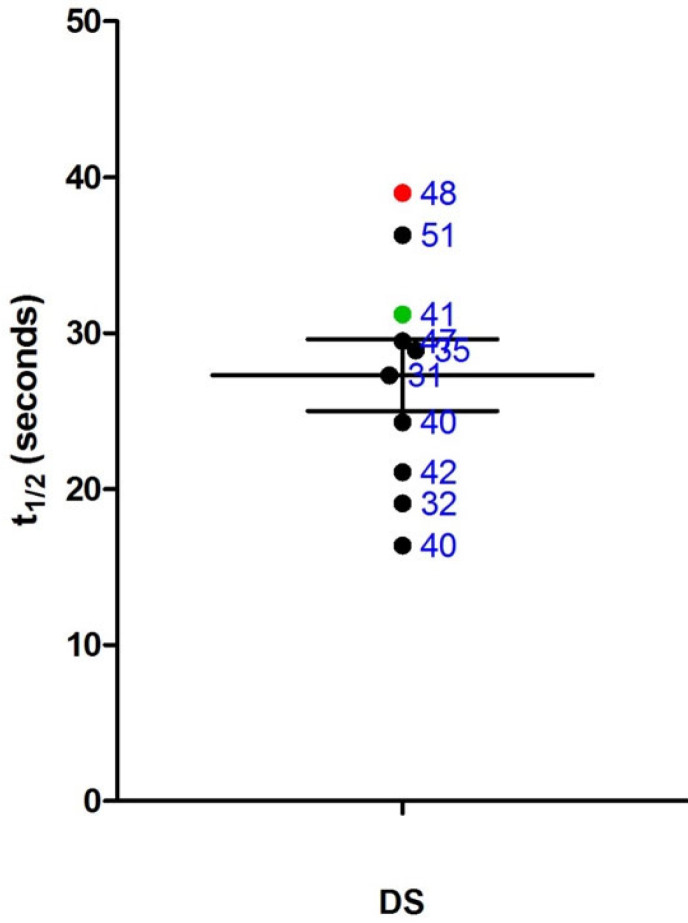
PCr recovery half-time (t_1/2_) in relation to APOE allelic status, with data points for Ɛ4 (red) and Ɛ2 (green) carriers labelled with individual ages in years. Each data point represents an individual participant.

**Figure 5:**
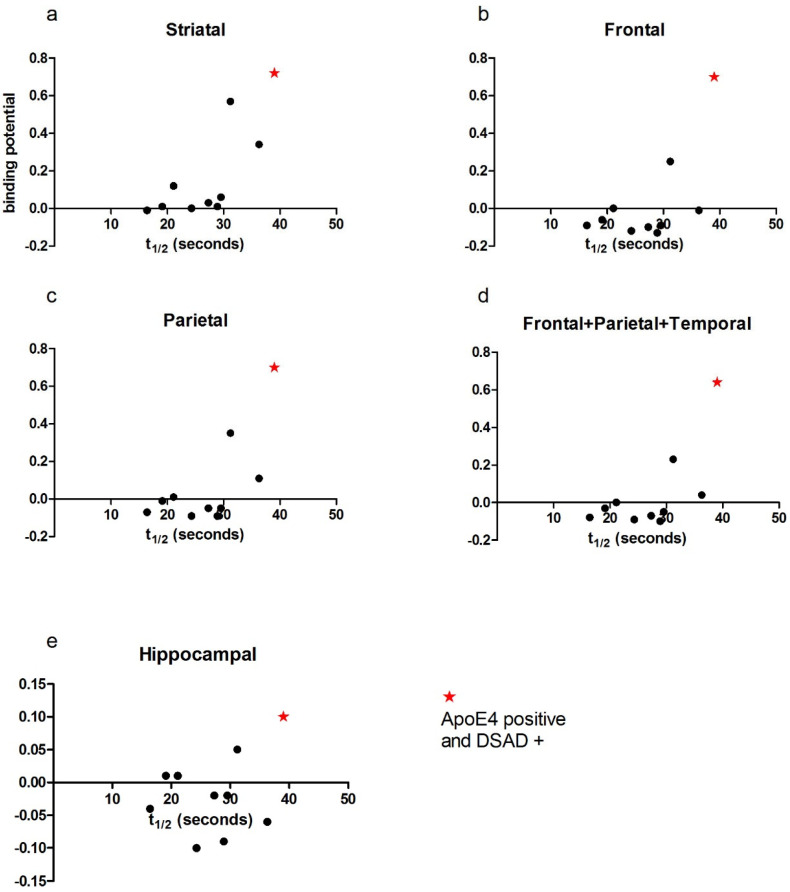
PCr recovery half-time (t_1/2_) correlations with amyloid binding data for the APOE Ɛ4 carrier, the only participant with dementia, highlighted across all analysed brain regions (**a**–**e**). Each data point represents an individual participant.

**Figure 6:**
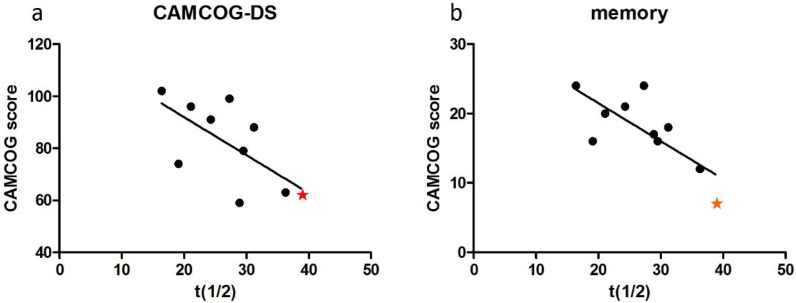
PCr recovery half-time (t_1/2_) correlations with cognitive performance data on the CAMCOG-DS overall score and the CAMCOG-DS memory domain for the APOE Ɛ4 carrier, the only participant with dementia (denoted by the star symbol) (**a**,**b**). Each data point represents an individual participant.

The participant with the Ɛ4 allele was also the only individual diagnosed with dementia in the cohort. This participant displayed the most mitochondrial dysfunction (highest PCr recovery half-time) (Figure 4) and the highest amyloid binding levels across all brain regions (Figure 5a–e). This individual also exhibited the poorest cognitive performance on the CAMCOG-DS (total score and memory domain scores) (Figure 6a,b).

## 4. Discussion

This pilot study provides the first in vivo evidence to suggest that mitochondrial dysfunction as measured in vivo in skeletal muscle plays a significant role in DSAD and age-related cognitive decline in adults with DS. We found that mitochondrial dysfunction (as measured by the PCr recovery half-time: a measure inversely proportional to mitochondrial oxidative capacity) in skeletal muscle correlates with cognitive performance metrics. Slowed PCr recovery is associated with increased amyloid-beta (Aβ) accumulation in key brain regions and with reduced functional outcomes in memory and executive functioning; these findings echo previous research in the general population and in mouse models suggesting that mitochondrial dysfunction is an important contributor to the DSAD disease process [2,3,4].

### 4.1. Mitochondrial Dysfunction and Amyloid-β Accumulation

We observed that in DS adults, greater mitochondrial dysfunction, evidenced by slowed PCr recovery, positively correlates with increased cortical Aβ accumulation, notably in the striatum—a region where amyloid deposition often initiates in both familial and DS-associated Alzheimer’s disease [43,44]. Previous studies in vitro and in mouse models have highlighted that mitochondrial Aβ accumulation disrupts key enzymes and elevates reactive oxygen species (ROS) production, leading to oxidative damage that further accelerates neuronal dysfunction [45,46,47]. Mechanistic studies have also revealed that Aβ oligomers interact with mitochondrial enzymes, exacerbating oxidative stress and membrane depolarization, which contribute directly to neuronal apoptosis and synaptic dysfunction [48,49]. The strong correlation observed between mitochondrial function and PiB binding in the striatum in this study presents the first in vivo human evidence in DS that amyloid burden and mitochondrial function are significantly correlated and therefore may influence one another in the ways indicated in the in vitro and mouse studies referenced above.

Linear regression analysis was applied to our data, as previous research in mouse models has indicated a linear relationship between mitochondrial function and amyloid binding in the brain [8]. Whilst we observed a strong positive correlation between amyloid binding and mitochondria in the striatum, the poor linear convergence observed in other regions could result from a number of factors (Figure 1). These include the small sample size presented in the current study as well as additional factors such as threshold effects, where low amyloid levels are tolerated but higher levels overwhelm compensatory mechanisms, and feedback loops, where amyloid-induced mitochondrial dysfunction increases reactive oxygen species presence and further exacerbates amyloid accumulation [5].

### 4.2. Mitochondrial Dysfunction and Cognitive Impairment

Our findings also correlate mitochondrial dysfunction with cognitive deficits, particularly in memory and executive functions, similar to those observed in Alzheimer’s and other neurodegenerative diseases [1,50]. This supports the “mitochondrial dementia” concept seen in primary mitochondrial disorders, where energy deficits lead to cognitive decline due to limited synaptic function and plasticity [50,51]. Our findings of strong correlations between mitochondrial dysfunction and memory and executive function scores align with evidence suggesting that mitochondrial stress in DS could exacerbate deficits in synaptic function, impacting energy-dependent cognitive processes [52].

The relationship observed here between mitochondrial dysfunction and memory impairment in DS adults aligns with animal studies that have indicated that mitochondrial dysfunction affects memory processes, particularly with high mitochondrial DNA (mtDNA) mutation loads [53]. It is possible that the high energy demands of memory formation and recall are compromised due to mitochondrial dysfunction, resulting in synaptic degradation and cognitive decline [54]. Together these findings suggest that mitochondria play an important and direct role in the aetiology of cognitive decline in adults with DS.

In the current study, mitochondrial impairment was more strongly associated with executive dysfunction than frontal amyloid deposition, as evidenced by a stronger correlation coefficient, suggesting a direct impact of mitochondrial health on frontal lobe function that does not necessarily involve just Aβ. Structural abnormalities are present in the frontal and temporal lobes in individuals with DS, suggesting a vulnerability to insult, [55] with frontal lobes being among the first to be compromised in DSAD [36]. A study of adolescents with DS has shown impaired executive function compared to TD children [56] and those with Williams syndrome [57], suggesting that impairments in executive function are a syndrome-specific characteristic of DS, potentially mediated by mitochondrial function. The high energy demands of frontal regions, driven by recurrent synaptic firing, make these areas particularly reliant on abundant and functional mitochondria [58,59]. In AD, synaptic mitochondrial dysfunction is one of the earliest detectable changes in both mouse models and human neurons, suggesting that a systemic mitochondrial defect could exacerbate cognitive decline by disproportionately affecting energy-dependent frontal regions [60,61,62].

### 4.3. APOE ε4 and Mitochondrial Dysfunction

One notable finding from this study was that the APOE ε4-positive participant exhibited the most pronounced mitochondrial impairment, cognitive decline, and amyloid burden. This individual was also the only participant with a confirmed diagnosis of DSAD. Although conclusions cannot be drawn from a single case, these results lend support to the hypothesis of a link between mitochondrial function and APOE Ɛ4 status. APOE, a 34-kDa protein involved in lipid transport and redistribution, plays a crucial role in mitochondrial assembly and energy production. Research suggests that APOE Ɛ4 fragments can interact with mitochondria, causing dysfunction and neurotoxicity [63].

In the general population, mitochondrial damage in AD appears to vary with APOE genotype [64]. In APOE Ɛ4 carriers, mitochondrial enzyme activity correlates more strongly with clinical dementia severity than with the densities of neuritic plaques or tangles. This contrasts with Ɛ4-negative patients in whom plaques and tangles better correlate with cognitive dysfunction [64]. Additionally, mitochondrial dysfunction may precede amyloid pathology in APOE Ɛ4 carriers, as young carriers have shown reduced mitochondrial function without significant amyloid or tau pathology [65]. It is plausible that early mitochondrial dysfunction in DS could exacerbate APOE Ɛ4′s detrimental effects, contributing to the early onset of AD.

However, our findings may challenge the APOE–mitochondria relationship in DSAD. The mitochondrial involvement of a protective effect of APOE Ɛ2 status does not seem to be supported as our APOE Ɛ2-positive participant was 8th out of 10 in terms of PCr recovery half-time, although the mechanisms through which Ɛ2 is protective are unknown and may differ to those mediating Ɛ4 influence. Larger, more controlled studies are necessary to clarify these interactions and their role in disease modification.

### 4.4. Limitations and Future Directions

This study provides new insights into the association between mitochondrial function and cognitive decline in DSAD, though our small sample limits the statistical power of some findings and the conclusions drawn. Moreover, the cross-sectional associations in this study cannot establish temporality or causation. It remains uncertain whether mitochondrial dysfunction initiates amyloid formation or arises as a result or byproduct of it. Future studies should involve larger, longitudinally followed DS cohorts to explore the causative pathways linking mitochondrial dysfunction to DSAD. Investigating the role of APOE and other mitochondrial genotypes more thoroughly, along with examining mitochondrial biomarkers over time and employing advanced neuroimaging techniques to assess mitochondrial function in the brain, would clarify the role of mitochondria in cognitive decline and aid in developing targeted interventions for DSAD prevention or delay.

## 5. Conclusions

Our study provides the first in vivo evidence of mitochondrial dysfunction as a potential driver of DSAD and cognitive impairment in DS. This study shows a positive association between slowed PCr recovery, amyloid burden, and cognitive decline, underscoring mitochondrial health as a crucial factor in neurodegeneration in people with DS. The mechanisms that underline the relationship between mitochondria and cognitive function remain speculative, and elucidation of such is beyond the scope of this manuscript. In the same vein, the nature of the mitochondria–Aβ interaction and the chronological ordering of mitochondrial dysfunction and the onset of amyloidosis in the natural history of DSAD are beyond the scope of this work. It is suggested, however, that mitochondrial dysfunction precedes amyloid deposition, as detected by PET, as the majority of the DS group exhibited little amyloid binding. Despite the small sample size, the relationships with both AD pathology and AD-related cognitive function add weight to the hypothesis that mitochondrial function is instrumental in DSAD.

## Figures and Tables

**Table 1:** Outline of tasks used in executive function battery [36,37,38,39,40,41,42].

Task	Processses Assessed	References
Tower of London	Planning, Working Memory	[36,37]
Weigl Sorting	Set Shifting, Abstraction	[36,38]
Verbal Fluency	Initiation, Set Shifting, Organization	[35,39]
Spatial Reversal	Set Shifting, Response Inhibition	[36,40]
Scrambled Boxes	Working Memory, Response Inhibition	[36,41]
Cats and Dogs	Response Inhibition, Rule Maintenance	[36,42]

**Table 2:** The characteristics of participants who completed both the DiDs and MRS studies.

**N**	10
**Sex**	8M, 2F
**Age range (y)**	31–51
**Age mean (±SD)**	41 ± 7
**IQ mean (±SD)**	54 ± 13

**Table 3:** APOE allelic frequencies among participants (N = 10).

	Ɛ2 Positive (Potential Protective)	Ɛ4 Positive (Potential Risk)	Ɛ3/3	Unknown
**n**	1	1	7	1

**Figure A1:**
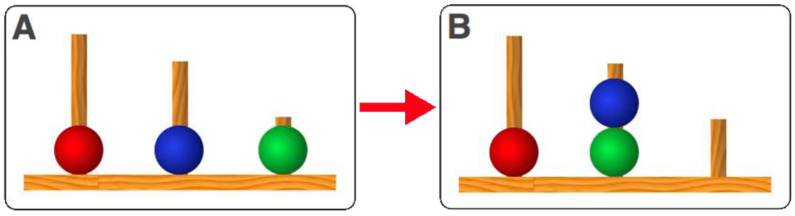
An example of the Tower of London task.

## Data Availability

The datasets presented in this article are not readily available due to technical/time limitations. Requests to access the datasets should be directed to J.B.W.

## Appendix A

### Appendix A.1. Tasks Used to Assess Executive Function

#### Appendix A.1.1. Tower of London Task

The Tower of London task measures planning ability and working memory using a peg board and three coloured beads. The aim is to move the beads to the correct position in the fewest number of moves whilst remembering to only move one bead at a time. Participants are given three attempts to complete the task and points awarded are reduced at each try.

#### Appendix A.1.2. Weigl Sorting Task

This task measures conceptual learning. The participant is presented with nine shapes: three circles, three squares, and three triangles. One of each shape is coloured red, blue, and yellow. Participants are asked to sort the items; correct sorts are by colour or shape. The items are then mixed up and the participant is asked to sort them a different way; to score maximum points, the participant must sort the shapes the other way first time.

#### Appendix A.1.3. Verbal Fluency Task

This task was taken from the CAMCOG test battery. Participants are instructed to verbally generate as many animals as possible within a one-minute period. The score is the total number of animals generated.

#### Appendix A.1.4. Spatial Reversal Task

This task measures spatial memory and response inhibition. At the start of the task, two coins are placed under two boxes, and the participant is asked to guess where the coins are. At this stage, the participant is always correct, and the coin is removed from whichever box the participant does not choose. The examiner then places a screen in front of the box and pretends to, but does not, move the coin. The participant needs to correctly find the coin, in the same place, four times before the coin is moved to the other box. At this stage, they are scored on consecutively choosing the correct (same) box four times.

#### Appendix A.1.5. Scrambled Boxes Task

This task measures working memory and response inhibition. To complete this task, the participant must correctly find three coins within three, and then six, boxes. Each box has a different shape on the top and the participant must attempt to not open boxes they have already checked and attempt to remember which boxes the coins have been placed in. In the first trial, the boxes stay in the same position; in the second trial, they are moved/scrambled by the examiner.

#### Appendix A.1.6. Cats and Dogs Task

The cats and dogs task measures response inhibition and working memory. Initially, participants are timed on naming cats and dogs correctly, and then training is given to name dogs as cats and cats as dogs. Responses are again timed and the difference between the first and second times is recorded.
